# Supplementary material for: A Spatially Coordinated Keratinocyte-Fibroblast Circuit Recruits MMP9+ Myeloid Cells to Drive IFN-I-Driven Inflammation in Photosensitive Autoimmunity
Source: bioRxiv. 2025 Aug 23:2025.08.19.670635. Preprint. [Version 1] doi: 10.1101/2025.08.19.670635 (PMC12393449; doi:10.1101/2025.08.19.670635)

bioRxiv preprint doi: https://doi.org/10.1101/2025.08.19.670635; this version posted August 24, 2025. The copyright holder for this preprint (which was not certified by peer review) is the author/funder, who has granted bioRxiv a license to display the preprint in perpetuity. It is made available under aCC-BY 4.0 International license.

Figure S1. Keratinocyte and lymphocyte subtypes characterization and molecular signatures

(A) Pseudo bulk expression heatmap of marker genes for main cell types and keratinocyte subtypes in scRNA-seq of blister biopsies. **(B)** Pseudo bulk expression heatmap of type I interferon (IFN-I)–responsive genes in lesional (L) and non-lesional

bioRxiv preprint doi: https://doi.org/10.1101/2025.08.19.670635; this version posted August 24, 2025. The copyright holder for this preprint (which was not certified by peer review) is the author/funder, who has granted bioRxiv a license to display the preprint in perpetuity. It is made available under aCC-BY 4.0 International license.

(NL) skin across diseases. **(C)** Box plots of protein levels (NPQ) in blister fluids across diseases and skins. T-test was used for pairwise comparisons: Not Significant (ns), $^*P < 0.05$, $^{**}P < 0.01$, $^{***}P < 0.001$) **(D)** Box plots showing percentage of activated keratinocyte (KC_activated) within all keratinocytes (KC) per sample across skin conditions. Statistical significance was assessed using scCODA, with KC_suprabasal as the reference cell type. **(E)** Pseudo bulk expression heatmap of canonical marker genes for lymphocyte subsets. **(G)** Box plots showing percentage of lymphocyte subtypes per sample across conditions. Statistical significance was assessed using scCODA, with KC_suprabasal as the reference cell type.

Figure S2. Cross-validation of myeloid subset composition and IFNB1 expression across scRNA-seq datasets in photosensitive skin diseases.

(A) Pseudo bulk expression of macrophage and dendritic cell defining marker genes in scRNA-seq of blister biopsies. **(B)** Box plots showing percentage of myeloid subsets within all myeloid cells (MC) per sample, across skin conditions. Each dot represents a sample. Statistical significance was assessed using scCODA, with KC_suprabasal as the reference cell type. **(C)** Pseudo bulk expression heatmap of myeloid cell marker genes across myeloid subsets in punch biopsies obtained from lesional skin of lupus (GSE179633). **(D)** Violin plot showing the expression of IFNB1 from myeloid subsets detected in blister biopsies. Each dot represents a single cell. **(E)** Pseudo bulk expression heatmap of myeloid cell marker genes across myeloid subsets in scRNA-seq of punch biopsies obtained from lesional skin of DM. **(F)** Violin plot showing the expression of IFNB1 from myeloid subsets detected in DM punch biopsies. Each dot represents a single cell.

Figure S3. Spatial transcriptomics reveals fine-grained subtypes and spatial organization of stromal, immune, and inflammatory cell states in autoimmune skin lesions.

(A-D) UMAP embeddings (left) and heatmaps for pseudo bulk marker genes expression of reclustered main cell types in seqFISH (A) Endothelial cells, (B) Fibroblasts, (C) Keratinocytes and (D) Lymphocytes. (E) Spatial embedding of seqFISH sections highlighting MC_CXCL8 cells using KC_granular as spatial reference. (F) Spatial embedding of seqFISH sections highlighting superficial proinflammatory fibroblasts (FB_proInf_sup), deep proinflammatory fibroblasts (FB_proInf_deep), and mesenchymal fibroblasts (FB_mesenchymal).

Figure S4. Spatial transcriptomics revealed transcriptional and spatial continuum along the LYVE1 to MMP9 transition.

(A) seqFISH data showing $CD14^+$ cells colored by their projection onto the first diffusion component in lesional tissue sections from the DLE and vitiligo samples. **(B)** Heatmap of differentially genes expressed in $CD14^+$ cells ordered by the first diffusion component (transition) ($p < 0.01$, tradeSeq) and aggregated into 30 bins (193 cells per bin). **(C)** Pseudo bulk expression heatmap of cytotoxic and Th1 markers in different

bioRxiv preprint doi: https://doi.org/10.1101/2025.08.19.670635; this version posted August 24, 2025. The copyright holder for this preprint (which was not certified by peer review) is the author/funder, who has granted bioRxiv a license to display the preprint in perpetuity. It is made available under aCC-BY 4.0 International license.

CD4 cell populations of the scRNA-seq data from blister biopsies. **(D)** Spatial embedding of seqFISH DM samples highlighting cytotoxic CD4_CXCL13 cell localization. **(E)** Representative spatial embedding illustrating radius used in colocalization analysis. **(F)** High-resolution image of CLE sample 2 showing zoom-ins of a dermal immune aggregate. Key cell types are displayed, with overlaid transcripts for LYVE1 (blue) and MMP9 (yellow).

Figure S5. (A) Heatmap of differentially genes expressed across the CD14+ cell transition in the GSE179633 dataset ($p < 0.01$, tradeSeq). Cells were ordered by the first diffusion component (transition) and aggregated into 50 bins (144 cells per bin). **(B)** As in 5F, but with ligand pseudo bulk expression computed using the GSE179533 data. **(C)** Scatter plot showing minimum distance in µm from a CD14+ cell to a superficial pro inflammatory fibroblast (x-axis) or to a deep pro inflammatory fibroblast (y-axis) colored by their projection onto the first diffusion component; marginal plots show mean of the first diffusion component of CD14+ cells projected onto the x-axis (bottom) or the y-axis (left).

Figure S6. (**A**) FACS gating strategy to quantify $CD14^+$ cells and myeloid cells in samples from non-lesional skin of a CLE patient. (**B-D**) Spatial embeddings highlighting relevant cell types in seqFISH data of the anifrolumab intervention experiment.

Figure S7. Type I interferon–licensed keratinocytes activate monocyte-derived dendritic cells (moDCs) and induce fibroblast-derived chemokine programs.

(A) Left: Gating strategy for flow cytometric analysis of moDCs. Right: Quantification of CD80 and HLA-DR expression in moDCs incubated with keratinocyte supernatants or directly stimulated with interferon-β (IFN-β) or lipopolysaccharide (LPS). Data are presented as mean ± standard error of the mean (SEM) and were analyzed using one-way ANOVA followed by Bonferroni post hoc test. **(B)** Top: Heatmap of protein expression in moDCs incubated with keratinocyte supernatants under the indicated UVB and interferon-β (IFN-β) treatment conditions, or directly stimulated with IFN-β or lipopolysaccharide (LPS). Bottom: Quantification of selected proteins from the heatmap, shown as mean ± standard error of the mean (SEM). Statistical analysis was performed using one-way ANOVA followed by Tukey's post hoc test. **(C)** Heatmap of selected inflammatory genes expression in bulk RNA-seq of moDCs stimulated with keratinocyte supernatants under the indicated UVB and interferon-β (IFN-β) conditions, or directly treated with IFN-β or lipopolysaccharide (LPS); colors are normalized expression scaled by gene (row). **(D)**

(H) Cytokine Concentration in keratinocyte supernatants, measured using OLINK proximity extension assay. Data are presented as mean ± SEM and were analyzed using one-way ANOVA followed by Tukey's multiple comparison test . (ns: not significant, * or # or ^ $p < 0.05$; ** or ## or ^^ $p < 0.01$, *** or ### or ^^^ $p < 0.001$, **** or #### or ^^^^ $p < 0.0001$).

Figure S1

bioRxiv preprint doi: https://doi.org/10.1101/2025.08.19.670635; this version posted August 24, 2025. The copyright holder for this preprint (which was not certified by peer review) is the author/funder, who has granted bioRxiv a license to display the preprint in perpetuity. It is made available under aCC-BY 4.0 International license.

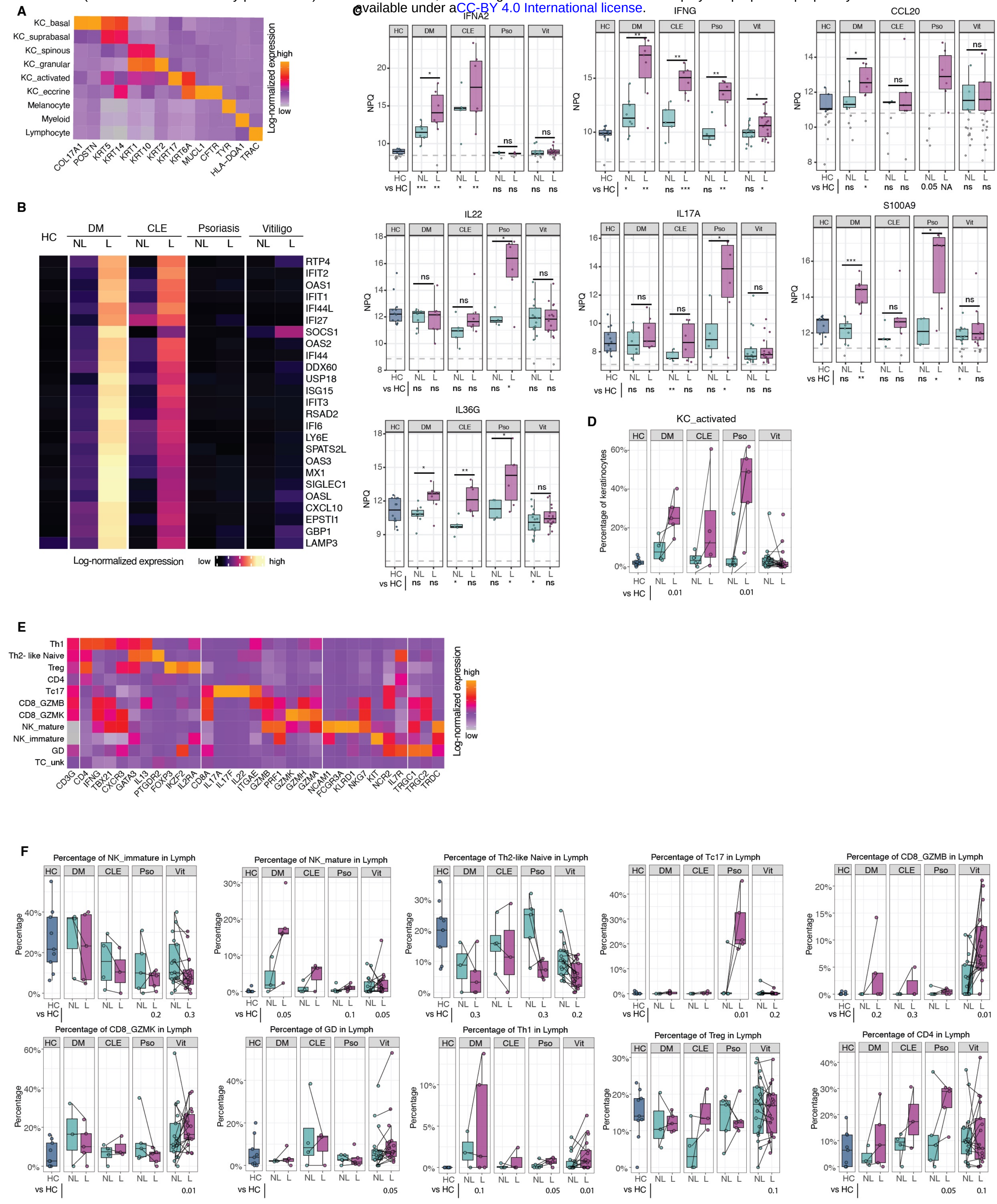

bioRxiv preprint doi: https://doi.org/10.1101/2025.08.19.670635; this version posted August 24, 2025. The copyright holder for this preprint (which was not certified by peer review) is the author/funder, who has granted bioRxiv a license to display the preprint in perpetuity. It is made available under aCC-BY 4.0 International license.

Figure S2

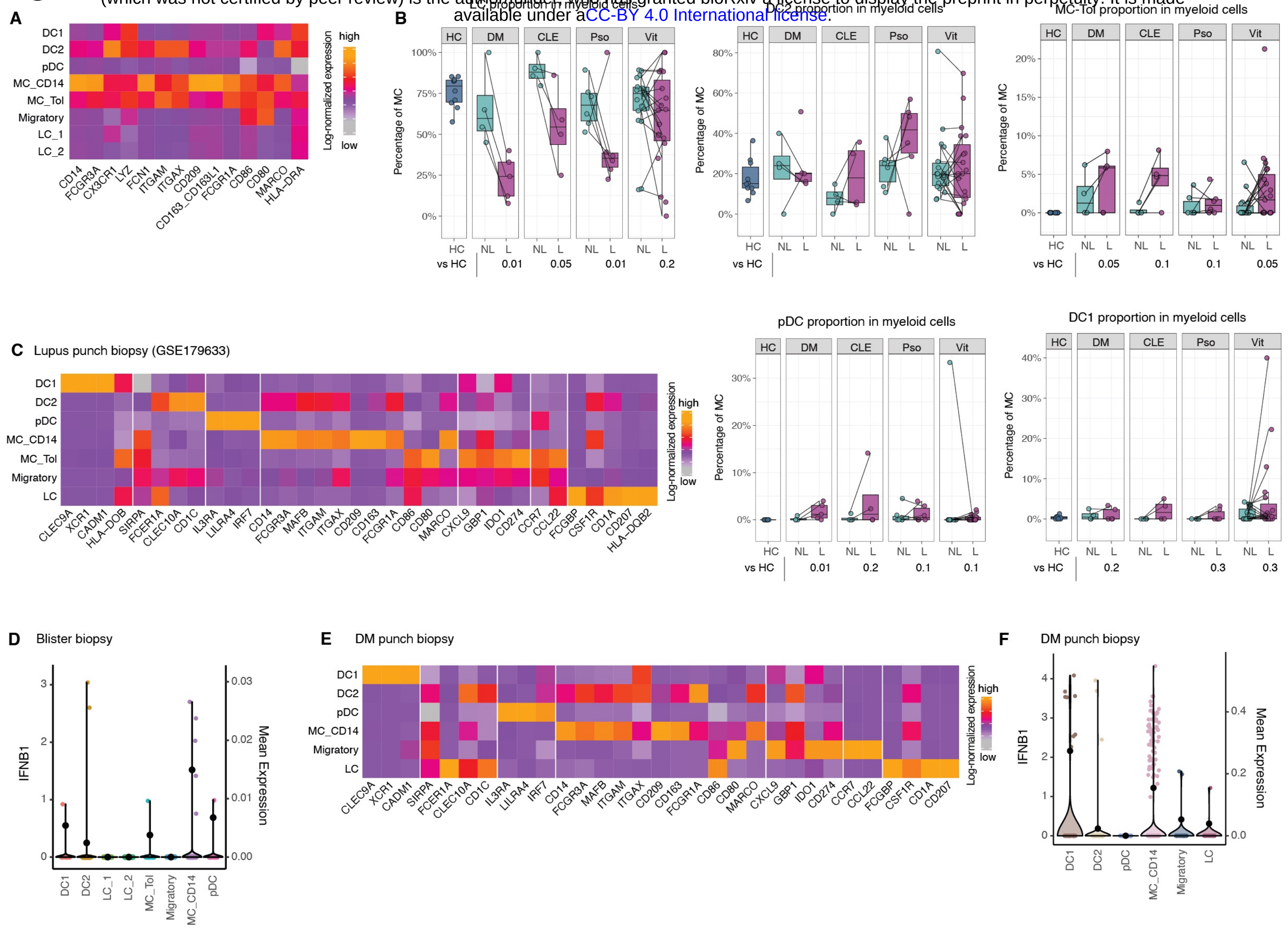

bioRxiv preprint doi: https://doi.org/10.1101/2025.08.19.670635; this version posted August 24, 2025. The copyright holder for this preprint (which was not certified by peer review) is the author/funder, who has granted bioRxiv a license to display the preprint in perpetuity. It is made available under aCC-BY 4.0 International license.

Figure S3

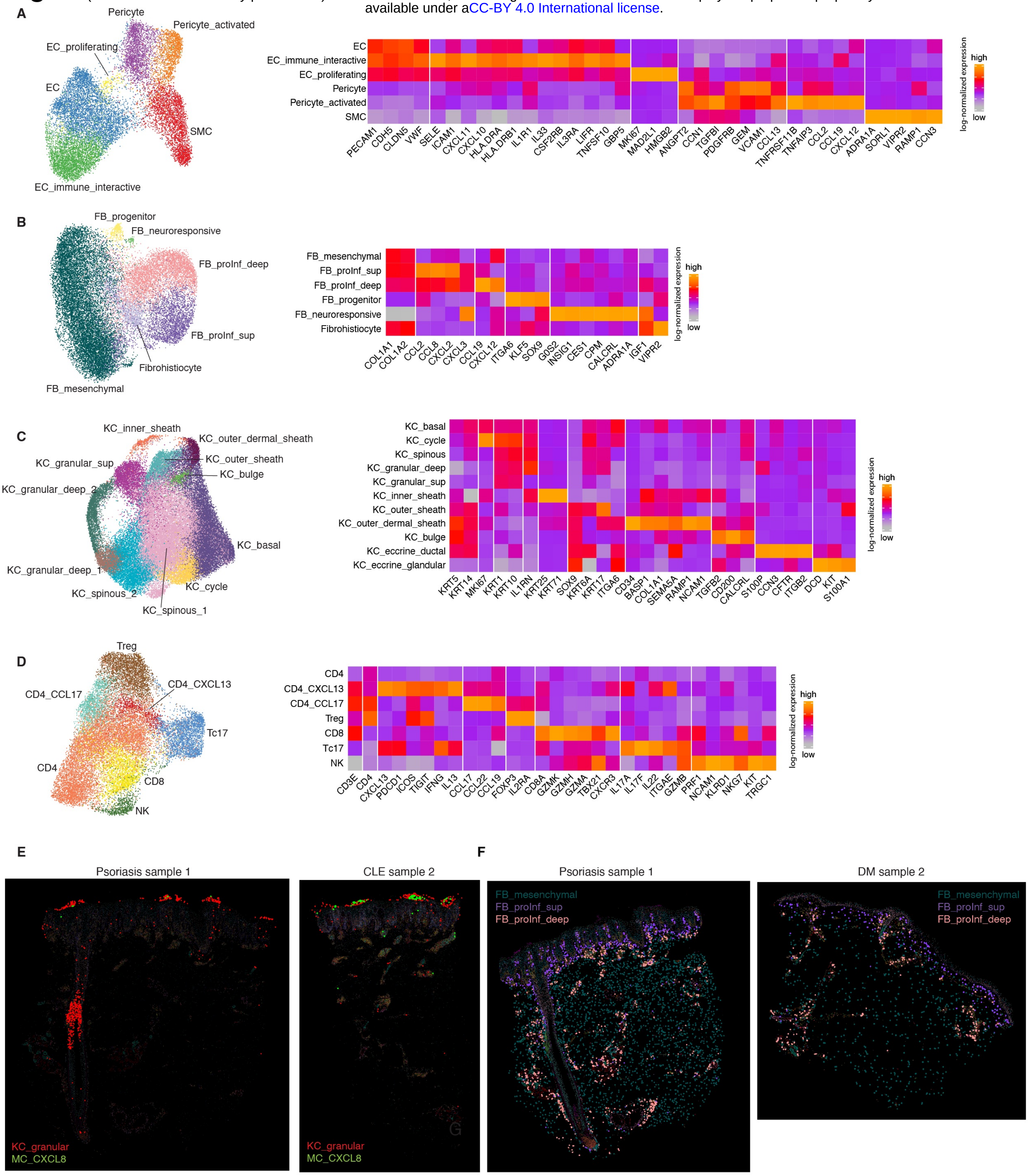

bioRxiv preprint doi: https://doi.org/10.1101/2025.08.19.670635; this version posted August 24, 2025. The copyright holder for this preprint (which was not certified by peer review) is the author/funder, who has granted bioRxiv a license to display the preprint in perpetuity. It is made available under aCC-BY 4.0 International license.

Figure S4

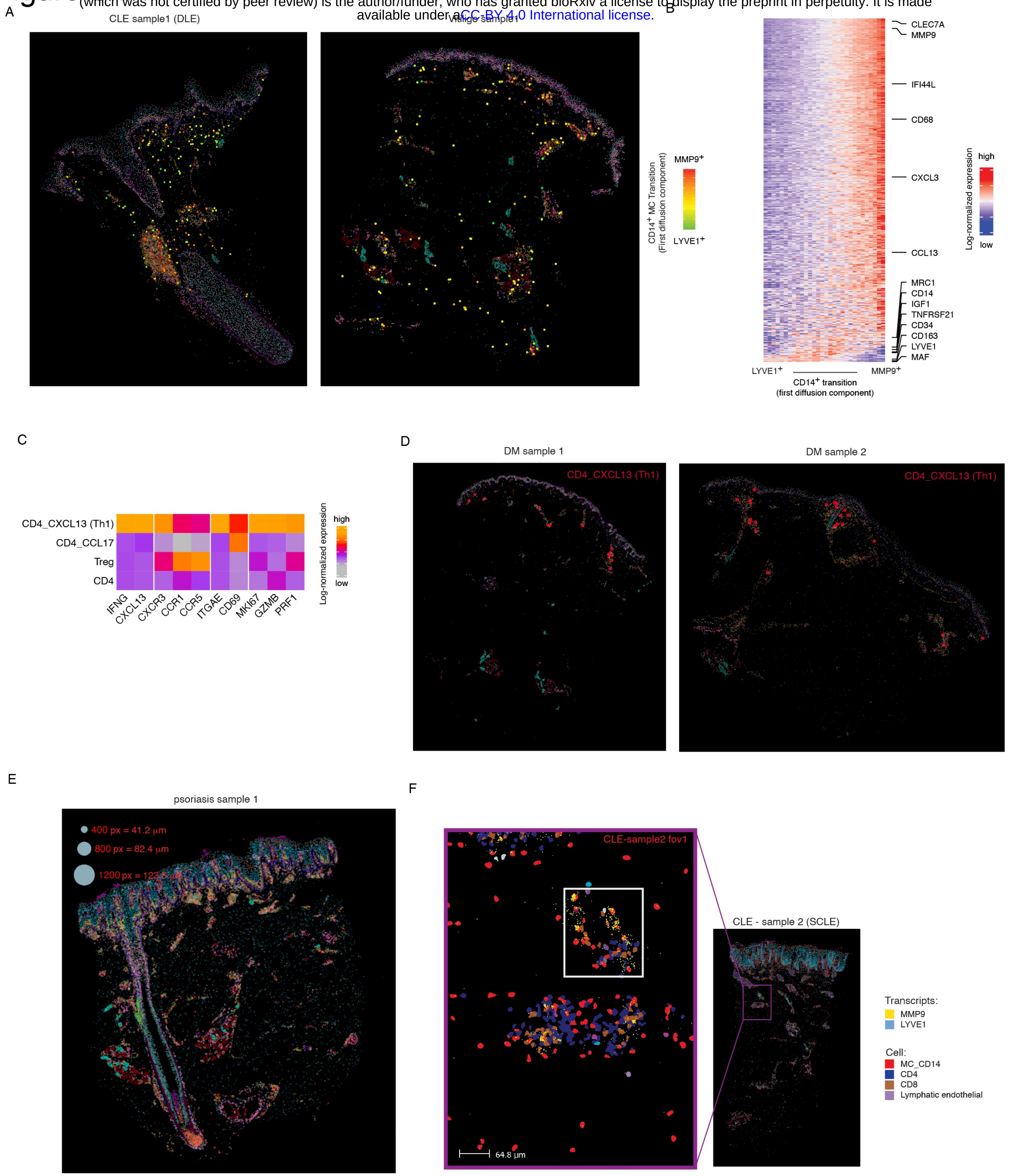

bioRxiv preprint doi: https://doi.org/10.1101/2025.08.19.670635; this version posted August 24, 2025. The copyright holder for this preprint (which was not certified by peer review) is the author/funder, who has granted bioRxiv a license to display the preprint in perpetuity. It is made available under aCC-BY 4.0 International license.

Figure S5

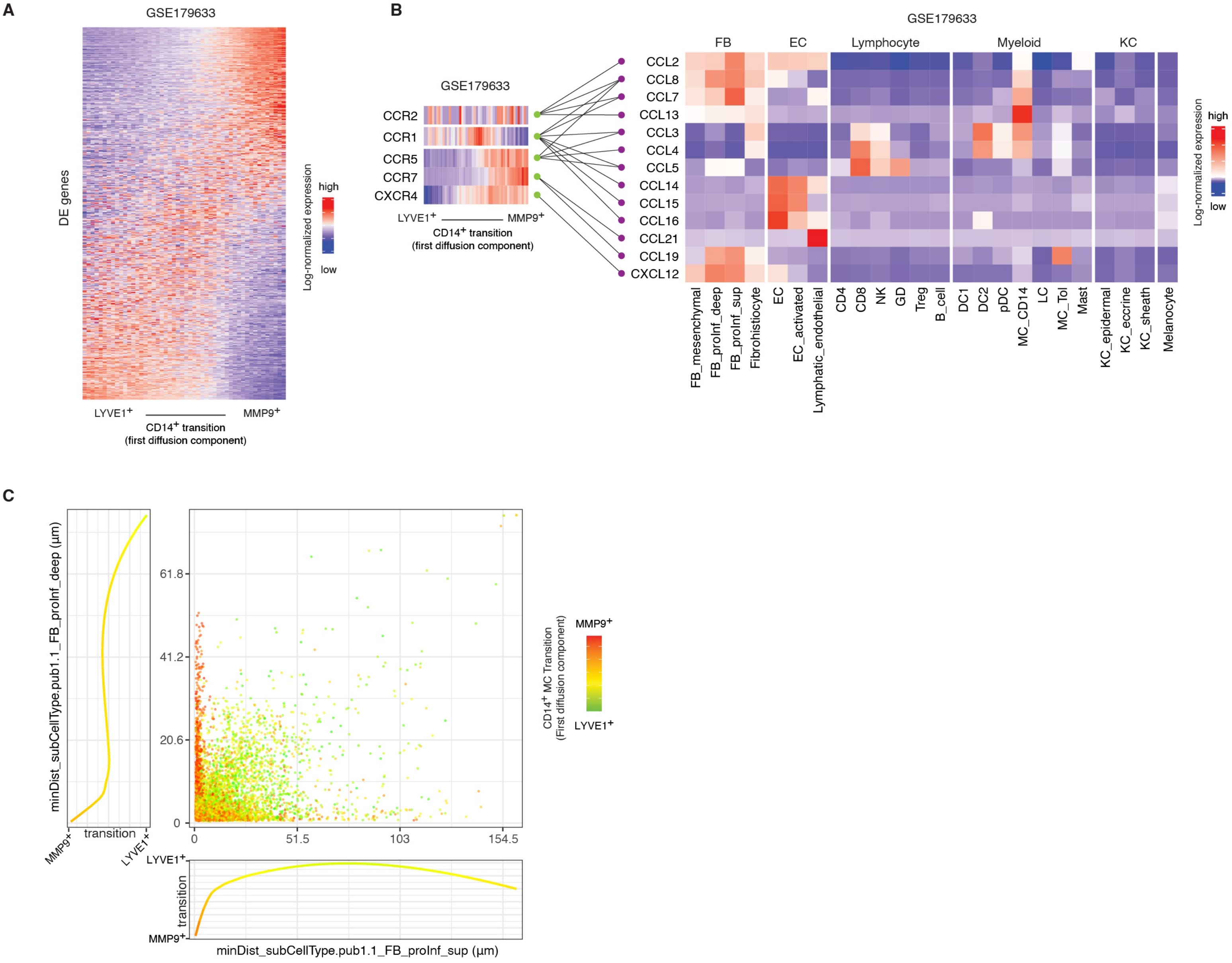

bioRxiv preprint doi: https://doi.org/10.1101/2025.08.19.670635; this version posted August 24, 2025. The copyright holder for this preprint (which was not certified by peer review) is the author/funder, who has granted bioRxiv a license to display the preprint in perpetuity. It is made available under aCC-BY 4.0 International license.

Figure S6

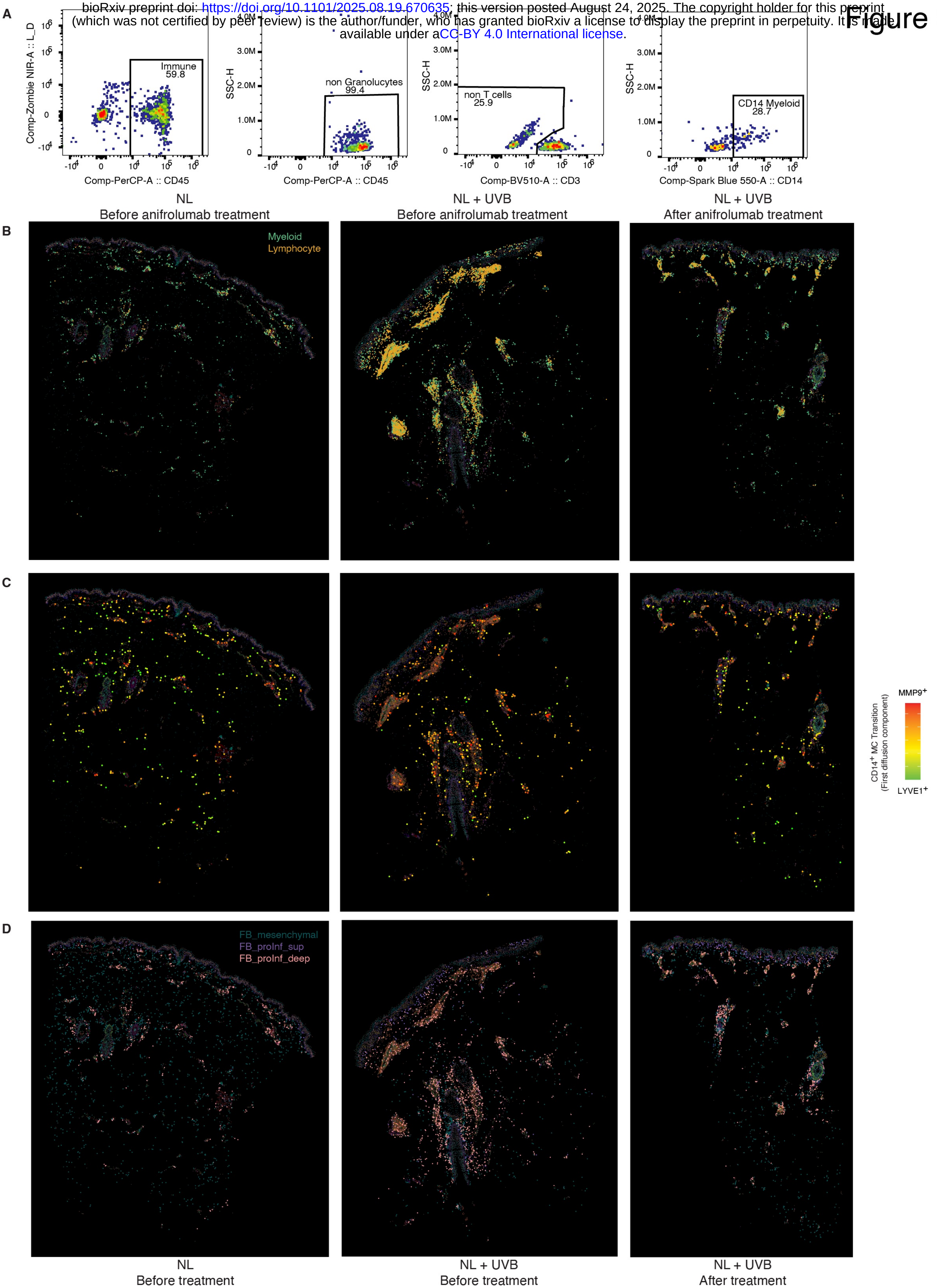

bioRxiv preprint doi: https://doi.org/10.1101/2025.08.19.670635; this version posted August 24, 2025. The copyright holder for this preprint (which was not certified by peer review) is the author/funder, who has granted bioRxiv a license to display the preprint in perpetuity. It is made available under aCC-BY 4.0 International license.

Figure S7

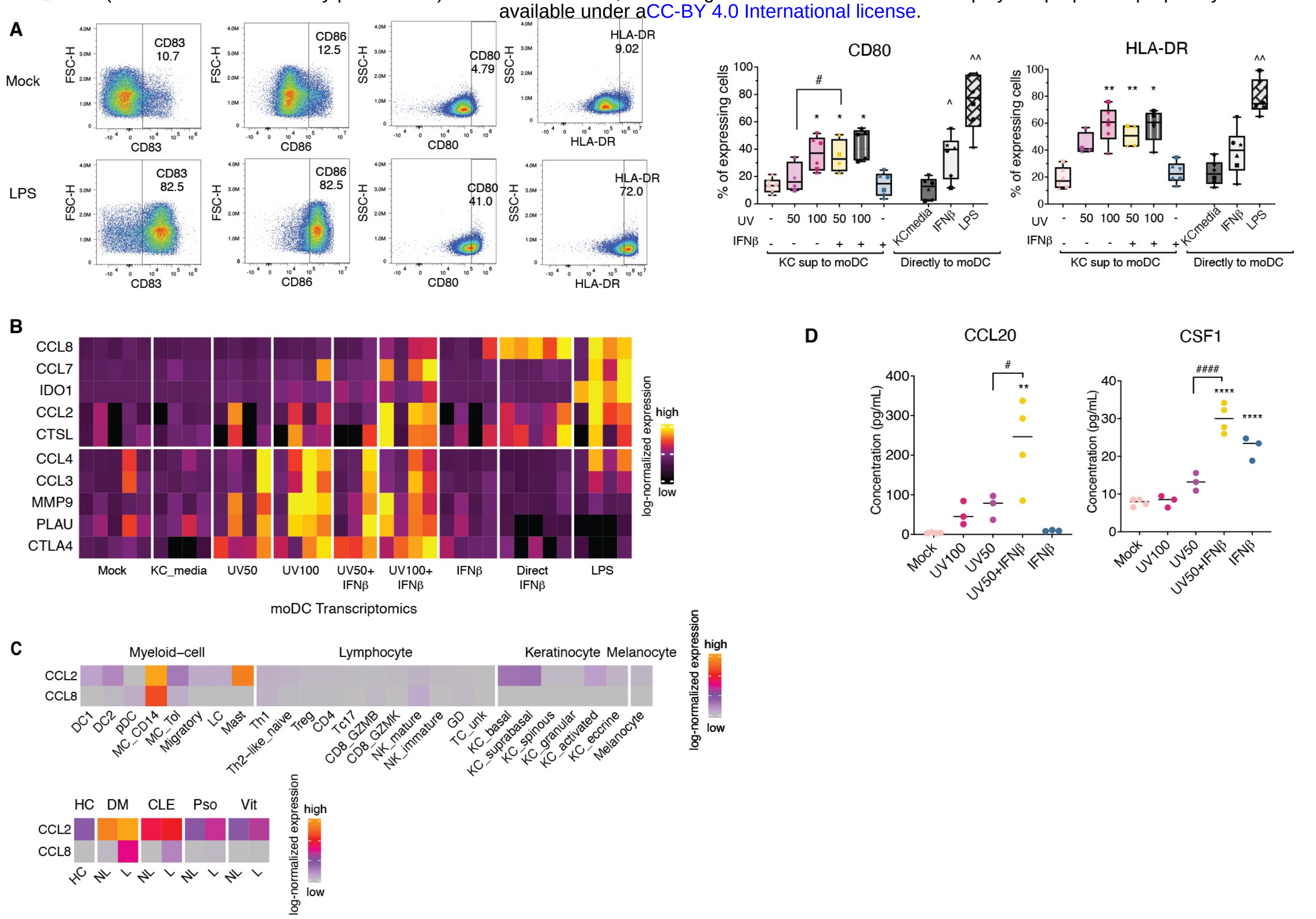

Supplement: 1 [file NIHPP2025.08.19.670635V1-supplement-1.pdf]
